# Psychological distress among women with abnormal pap smear results in Serbia: Validity and reliability of the Cervical Dysplasia Distress Questionnaire

**DOI:** 10.1371/journal.pone.0218070

**Published:** 2019-06-12

**Authors:** Irena Ilic, Goran Babic, Aleksandra Dimitrijevic, Milena Ilic, Sandra Sipetic Grujicic

**Affiliations:** 1 Faculty of Medicine, University of Belgrade, Belgrade, Serbia; 2 Department of Gynecology and Obstetrics, Faculty of Medical Sciences, University of Kragujevac, Kragujevac, Serbia; 3 Department of Epidemiology, Faculty of Medical Sciences, University of Kragujevac, Kragujevac, Serbia; 4 Institute of Epidemiology, Faculty of Medicine, University of Belgrade, Belgrade, Serbia; University of California Los Angeles, UNITED STATES

## Abstract

**Background:**

Receiving report of an abnormal finding from a Pap screening test in women often leads to psychological distress.

**Objectives:**

The purpose of this study was to assess the reliability and validity of the Cervical Dysplasia Distress Questionnaire (CDDQ) among women in Serbia.

**Methods:**

In 2017, we conducted a cross-sectional study involving 154 consecutive women attending cervical cancer screening who had received abnormal Pap smear results.

**Results:**

Reliability assessment showed good internal consistency for all CDDQ subscales (Tension and discomfort: Cronbach’s α = 0.844; Embarrassment: α = 0.864; Sexual and reproductive consequences: α = 0.867; and Health consequences: α = 0.913). The test-retest reliability showed that the correlation coefficients (between 0.805 and 0.983) were significant at the 0.01 level for all of the Serbian CDDQ subscales. Principal Axis Factoring with Direct Oblimin rotation indicated four main components that explain 55.0% of variance.

**Conclusion:**

The Serbian version of the CDDQ scale is a valid and reliable instrument for the assessment of psychological distress among women with abnormal Pap smear results.

## Introduction

A substantial decline in cervical cancer mortality rates has been noted following the introduction of high-coverage high-quality screening programs in developed countries [1]. In Serbia, as in other countries with limited resources, less than a third of cervical cancer cases are discovered in the early stage of illness, whilst in most patients later stages are found [2–4]. Achieving high rates of screening coverage is a challenge for both developing and developed countries.

Receipt of a report of abnormal finding from a Pap screening test in women often leads to withdrawal from participation in further monitoring and follow-up testing [5]. One of the reasons for nonadherence to follow-up after an abnormal Pap test result is psychological distress [6]. Psychological distress is defined as a state of emotional suffering characterized by symptoms of depression (e.g., sadness, hopelessness) and anxiety (e.g., restlessness, feeling tense) [7]. The main limitations of some of the previous studies that have been conducted with the objective of assessing psychological distress in women with abnormal Pap smear results include the use of general health questionnaires, non-validated questionnaires, or small study samples [8]. So far, only a few specific questionnaires have been developed for the assessment of psychological distress in women with abnormal cervical cancer screening results: the Psychosocial Effects of Abnormal Pap Smears Questionnaire (PEAPS-Q) [9], the Cervical Dysplasia Distress Questionnaire (CDDQ) [10], the Process and Outcome Specific Measure (POSM) [6]. Considering that the POSM is a questionnaire that was used in research in women with borderline and other low-grade abnormal smears and taking into consideration that the screening program in Serbia predicts that all women with abnormal Pap results are referred to further procedures (colposcopy, etc.), the CDDQ was the appropriate scale for the measurement of psychological distress.

In 2004 in the United States of America, Shinn et al [10] developed the CDDQ as an instrument for measuring the perception of diagnostic procedures and distress in women with positive screening tests for cervical cancer. The basis for the development of CDDQ was the PEAPS-Q, a scale developed in 1995 by Bennetts et al [9] for measuring psychosocial distress experienced by women undergoing follow-up investigation after an abnormal Pap smear result in Australia. The PEAPS-Q consists of 14 items and 4 factors: experience of medical procedures, beliefs and changes in perception of oneself, worry about infectivity and effect on sexual relationships. The PEAPS-Q is a validated scale, with good internal consistency (α = 0.84) and high test–retest reliability (*r* = 0.88) [9]. In order to make it more understandable for American and Canadian women, Shinn et al [10] significantly modified the PEAPS questionnaire and subsequently developed the CDDQ scale through 3 phases with 3 separate samples consisting of 661 women undergoing colposcopy after an abnormal Pap smear finding: phase I (the PEAPS-Q was administered to 253 colposcopy patients, and 20 new items were generated), phase II (the modified PEAPS-Q was administered to a new sample of 89 colposcopy patients, and 2 of the new items were dropped since they were not easily understood by patients), and phase III (the modified items were administered to a new sample of 319 patients undergoing colposcopy, and there were no further concerns about the interpretability of the CDDQ items). Finally, the CDDQ scale contains 23 items (11 items remained from the PEAPS-Q scale, while 12 new items were added) that are divided into 2 sets: distress during medical procedures (resulting in 2 factors—embarrassment and discomfort/tension), and distress items (resulting in 2 factors—sexual and reproductive issues, and health consequences).

The original CDDQ subscales had good internal consistency (ranging from 0.76 to 0.90) and showed good concurrent validity with psychometrically validated measures of distress (i.e. Center for Epidemiological Studies Depression Scale, Spielberger State-Trait Anxiety Scale, Cancer Worry Scale, and the single-item Pain and Anxiety Rating Scales) [10]. Two of the CDDQ subscales in particular—sexual and reproductive consequences, and health consequences regarding the procedures—demonstrated good internal consistency reliability (α = 0.85 and α = 0.92, respectively) in a study undertaken to evaluate a strategy for improving follow-up of an abnormal Pap test in low-income and minority women [11]. Unfortunately, the CDDQ scale has had limited implication in empirical studies because it measures domains of distress experienced specifically after colposcopy or gynaecological examination, so that its applicability in women undergoing different kinds of management, such as cytological surveillance only, is unknown [12, 13]. A parallel system of either organized or opportunistic cervical screening with the Pap test has been ongoing in all regions of Serbia for the past few years [12]. The policy underlying this nationwide practice cites several tests in its recommendations, including gynaecological examination, speculum examination, Pap test and routine colposcopy. While colposcopy is often carried out even when a Pap test is done, the screening protocol does not necessitate both of these procedures in tandem [12].

Although the CDDQ scale was developed in English, it has not been widely translated. Even though in the available literature there is no data on its psychometric characteristics, there is a German version of CDDQ with three subscales—tension and discomfort, embarrassment during a gynaecological examination, and concerns about sexual and reproductive consequences—with use reported in women diagnosed with human papilloma virus-related precancerous genital lesions [14]. One subscale (health consequences) was omitted in the German version of CDDQ, because investigators believed that the items were inappropriate for the study population. The Serbian version developed in this study, is reported here for the first time.

Serbia has seen high mortality rates and an unfavourable trend in mortality of cervical cancer over the past decades, largely due to the lack of Pap smear screening [15]. Although the population-based screening for cervical cancer was introduced in 2013 [2, 16], the practical realization of this national program has been challenging. For example, a more standardized approach to investigating psychological distress in women with abnormal screening results for cervical cancer is needed. Identification and education of women who are at the highest risk of psychological distress during the cervical cancer screening would enable greater coverage by the medical procedures. Today, there remains a dearth of questionnaires specifically for cervical cancer screening or related medical procedures, such as Pap test, colposcopy, etc. Moreover, while research on the quality of life in Serbian women with cervical intraepithelial lesions who were treated by single cervical excision has been published [17], studies of the psychological effects of diagnostic procedures remain lacking. The aims of this study were to evaluate the internal consistency (internal reliability), test-retest reliability (external reliability) and to assess factor structure (construct validity) of the Serbian version of the CDDQ scale among women with abnormal Pap smear result.

## Methods

### Study setting

This study was conducted at the Clinical Center Kragujevac (Serbia), one of the four health institutions in the country that provides tertiary health care. As such, it is one of the main health institutions responsible for carrying out the national screening program for cervical cancer in Kragujevac [17].

### Study design

A cross-sectional study design was used, with analyses based on the collection of self-reported data. Questionnaires were distributed to all participants prior to their diagnostic examinations, along with a cover letter outlining the study’s information and a written consent form for study participation.

### Study sample

Among the target group of women for cervical cancer screening (age range of 25–64 years-old), every consecutive woman who received abnormal Pap smear results and had undergone the subsequent diagnostic examinations was included in the study.

Eligible women were aged 25–64 years, had had a Pap smear taken routinely as a part of the population screening program in the previous one year that showed an abnormality, were residents in the Kragujevac district area, and were fluent in spoken and written Serbian language. Women were ineligible if they were illiterate in Serbian language, had a Pap smear done more than one year ago, refused diagnostic examination, refused to participate in the study, were aged < 25 years or > 64 years, were pregnant at the time of recruitment, had had previous treatment for cervical lesions, or had neuropsychiatric disorders that would hinder participation in the study. Among the 190 eligible women, the study sample included a total of 154 women (participation rate: 81.1%). Reasons for ineligibility or refusal to participate were documented, with the latter including vision disorders, lack of time for participation, and lack of interest in the study. Questionnaires that were not fully completed were excluded from the analysis.

### Sample size calculation

The sample size necessary for factor analysis was estimated based on the recommendations provided by Mundfrom et al [18]. Considering the ratio of variables to factors as 5.75 and number of factors being 4, the minimum necessary sample size for conducting factor analysis with a 0.92 coefficient of congruence ranged from 55 to 130. Coefficients of congruence of 0.92 or greater are considered to reflect good to excellent matching, based on the guidelines given by MacCallum et al [19]. Therefore, we planned our minimum sample size to be 130.

### Data collection

Women eligible for study were asked to complete a socio-demographic and lifestyle questionnaire and the CDDQ questionnaire. Participants were allotted approximately 20 (±5) minutes to complete the questionnaires. Additionally, the re-test was conducted approximately 2–4 weeks after diagnostic procedures at the Clinical Centre in Kragujevac.

### Study timepoints

The ‘initial visit’ to the Clinic of Gynecology and Obstetrics at the Clinical Center Kragujevac when women with abnormal Pap smear results were addressed by the staff and entered into the study represented time-point 1. At this visit, participants provided voluntary informed written consent, completed the “initial” survey (CDDQ—testing), and underwent the diagnostic procedures (colposcopy/biopsy/endocervical curettage). All diagnostic procedures were implemented according to the criteria set out in the procedures of the National Guide for Good Practice in the Diagnosis and Treatment of Cervical Cancer in Serbia [20] and performed by a gynaecology specialist doctor.

The next study time-point (time-point 2) corresponded to the moment immediately before the women received their results from the diagnostic procedures performed, representing an interim period of two to four weeks from time-point 1. The participants filled out the questionnaire (CDDQ—retesting) under the same conditions, in the presence of a doctor or a nurse who were available to address any difficulties in the women’s understanding of certain issues. All participants attended the test-retest reliability.

### Instruments of data collection

The socio-demographic and lifestyle questionnaire obtained data on age, place of residence, educational level, occupation, marital status, menarche, menopause, pregnancy, oral contraceptive use, family history of cervical cancer, tobacco and alcohol use, etc. The CDDQ obtained data related to distress regarding the medical procedures and the consequences of receiving an abnormal Pap smear result in the past 12 months. The CDDQ is a self-report 23-item scale comprising the four subscales of Tension and discomfort (6 items), Embarrassment (2 items), Sexual and reproductive consequences (9 items), and Health consequences (6 items). Participants’ degree of agreement with items was scored on a 4-point scale, with the response options of 1 (Not at all), 2 (Somewhat), 3 (Moderately so), and 4 (Very much so) [10]. The total score was obtained by averaging the scores of the all items, with a higher score denoting a higher level of psychological distress.

Translation and cultural adaptation of the CDDQ for Serbia were performed in accordance with the internationally accepted methodology [21]. The ‘forward’ translation (from English to Serbian) was followed by a ‘backward’ translation. Initially, the forward translation was carried out by two bilingual translators working independently, both being native Serbian speakers. Following that, the two translators met to compare their translations, resolve differences, and agree on a common version of the forward translation which was then evaluated by a set of experts (translators and health professionals). The translated CDDQ was then reviewed by a focus group (gynaecologist, medical doctor—junior researcher, nurse, women undergoing colposcopy many times over the past years), after which a bilingual professor, whose mother tongue is English, performed the backward translation from Serbian to English. The resultant Serbian version of the CDDQ was then tested in a pilot study that involved five women in the target population (who attended cervical cancer screening) and which showed that participants did not encounter difficulties with comprehension of items or with completing the questionnaire. None of the respondents stated any remarks to the questionnaire, either during or after the survey.

### Data analyses

Based on the Classical Test Theory, two approaches were used to assess the reliability of the CDDQ: internal consistency reliability (measured with Cronbach’s α coefficient) and test-retest reliability (quantified with a correlation coefficient) for each dimension. A Cronbach’s α coefficient value of ≥ 0.7 was considered acceptable but values ≥ 0.8 were preferred. Test-retest reliability coefficients vary between 0 and 1, where values of ≥ 0.8 indicate good reliability but values ≥ 0.9 denote excellent reliability. The time-point 1 data was used for evaluation of internal consistency and validity, while both time-point 1 and time-point 2 data was used for the assessment of test-retest reliability. In order to determine the validity of the CDDQ, construct factor analysis (Principal Axis Factoring) was performed. The Kaiser-Meyer-Olkin measure of sampling adequacy was 0.804. Bartlett's test of sphericity was highly significant (*p* < 0.001). Factors were extracted using the Principal Axis Factoring method, followed by oblique rotation of the factors using Direct Oblimin rotation with Kaiser Normalization (delta = 0). The factors’ importance was considered according to the Kaiser criterion. Plausible factors were extracted using the Kattel's scatter diagram. Additionally, the contribution of each of the scale items to the discriminating ability was determined by the item-total correlation, where the items with item-total correlation > 0.20 were regarded as acceptable [22].

All statistical analyses were conducted using the Statistical Package for Social Sciences Software (SPSS Inc, version 20, Chicago, IL).

### Ethical considerations

This study is a part of a research project approved by the Ethics Committee of the Faculty of Medical Sciences, University of Kragujevac (Ref. No.: 01–2176) and by the Ethics Committee of the Clinical Center Kragujevac (Ref. No.: 01–2869).

## Results

### Participant characteristics

The majority of participants were aged < 55 years, with urban residence and higher educational level (Table 1). Also, most of the women reported having a current partner and having been pregnant at least once in their lifetime. Abortion experience was reported by 68.8% of the women. Oral contraceptive use was reported by 22.7% of women. About half of the women were premenopausal at the time of this study. Family history of cervical cancer was reported among 11.0% of the women. More than half of the participants reported ever use of tobacco.

**Table 1:** Demographic characteristics of study participants variable.

	Number (n = 154)	%
**Age** (< 55 years)	101	65.6
**Place of residence** (Urban)	128	83.1
**Educational level** (> 8 years)	133	86.4
Occupation* (Manual worker)	64	41.6
**Marital status** (With partner)	120	77.9
**Pregnancy** (Yes)	147	95.5
**Children** (Yes)	146	94.8
**Menopause** (Yes)	74	48.1
**Oral contraceptive use** (Yes)	35	22.7
**Abortion history** (Yes)	106	68.8
**Family history of cervical cancer** (Yes)	17	11.0
**Tobacco use** (Ever)	90	58.4
**Alcohol use** (Yes)	21	13.6

* For retiree the occupation before retirement was shown.

### Reliability

The reliability of the Serbian CDDQ questionnaire had high internal consistency (Table 2). The Cronbach’s α coefficients for the CDDQ subscales were 0.844 for Tension and discomfort, 0.864 for Embarrassment, 0.867 for Sexual and reproductive consequences, and 0.913 for Health consequences. All of the items correlated well with the average of the others, with all items exceeding the acceptable level for the item-total correlation coefficient of 0.20.

**Table 2:** Internal consistency reliability of CDDQ among women with abnormal Pap smear results in one University hospital in Serbia.

CDDQ	Mean	Standard deviation	Item-Test Score Correlation	Cronbach's Alpha if Item Deleted	Cronbach’s alpha coefficient
**Tension and discomfort**					**0.844**
Did you find the exams uncomfortable?	1.78	0.794	0.564	0.832	
Did you find the exams emotionally upsetting?	1.82	0.889	0.663	0.813	
Did the exams make you nervous?	2.15	1.015	0.662	0.814	
Did the exam hurt?	1.56	0.775	0.470	0.847	
Did you feel tense?	2.09	0.945	0.721	0.801	
Were you nervous?	2.06	0.934	0.682	0.809	
**Embarrassment**					**0.864**
Were you uncomfortable being partly undressed?	1.97	0.983	0.760	0.880	
Were you embarrassed having your private parts touched by the doctor or nurse?	1.71	0.900	0.760	0.880	
**Sexual and reproductive consequences**					**0.867**
How worried are you that you would lose your chance to have a baby?	1.68	0.722	0.347	0.872	
Have you been worried that you could give the problem to a sexual partner?	1.88	0.979	0.480	0.866	
Have you been worried whether a sexual partner will think they can catch the problems from you?	1.88	0.900	0.764	0.834	
Have you been worried whether you should continue having sex?	1.70	0.785	0.657	0.846	
Have you been worried that this problem might affect how attractive you are to your sexual partner?	1.78	0.810	0.607	0.850	
Have you been worried whether having sex will make the problem worse?	1.47	0.716	0.610	0.851	
Have you been worried whether others think you have had more sexual partners than you should?	1.71	0.738	0.623	0.849	
Have you been worried about sex being more painful now?	1.76	0.841	0.645	0.846	
Have you been worried that this problem might affect how much you enjoy sex?	1.62	0.777	0.671	0.844	
**Health consequences**					**0.913**
How worried are you that cancer will appear in your body?	2.60	1.012	0.755	0.898	
Have you worried about the test results?	2.80	0.986	0.648	0.912	
Have you worried that you may have cancer?	2.41	1.082	0.742	0.900	
How worried are you that you might die?	2.47	1.080	0.808	0.891	
Have you been worried that your problem may turn into cancer?	2.71	1.047	0.812	0.890	
How worried are you that you might die from cervical cancer?	2.64	1.064	0.779	0.895	

CDDQ = The Cervical Dysplasia Distress Questionnaire.

The test-retest reliability coefficients were 0.805, 0.808, 0.959, and 0.983 respectively for the subscales of Tension and discomfort, Embarrassment, Sexual and reproductive consequences, and Health consequences (Table 3). The test-retest reliability showed that the correlation coefficients were significant at the 0.01 level for all of the Serbian CDDQ scores. The test-retest reliability coefficients for all four subscales (Tension and discomfort, Embarrassment, Sexual and reproductive consequences, and Health consequences) showed good stability of the tool.

**Table 3:** Test-retest reliability of CDDQ among women with abnormal Pap smear results in one University hospital in Serbia.

CDDQ domains	Tension and discomfort - REPEATED	Embarrassment - REPEATED	Sexual and reproductive consequences–REPEATED	Health consequences –REPEATED
**Tension and discomfort**	0.805*			
**Embarrassment**		0.808*		
**Sexual and reproductive consequences**			0.959*	
**Health consequences**				0.983*

* P<0.01.

CDDQ = The Cervical Dysplasia Distress Questionnaire.

### Validity

**Fig 1:**
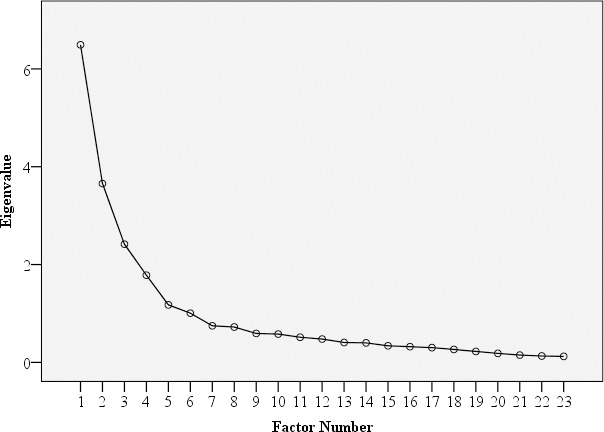
Screeplot of factor in the Cervical Dysplasia Distress Questionnaire.

The results obtained from the Principal Axis Factoring with Direct Oblimin rotation illustrated the presence of four main components with an Eigenvalue > 1, explaining 55.0% of variance (26.5%—Health consequences, 13.9%—Sexual and reproductive consequences, 8.7%—Tension and discomfort, and 5.9%—Embarrassment, respectively). The four components and factor loadings (with parts of variance explained) showed good Serbian CDDQ construct validity (Table 4). Inspection of the scree plot supported a four-factor solution (Fig 1).

**Table 4:** Factor analysis with Direct Oblimin Rotation Method for CDDQ items: Matrix of factor weights.

CDDQ items	Factor weights				Part of variance explained
	Component				
	Health consequences	Sexual and reproductive consequences	Tension and discomfort	Embarrassment	
Did you find the exams uncomfortable?	0.264	-0.020	**0.612**	0.354	0.448
Did you find the exams emotionally upsetting?	0.237	0.035	**0.696**	0.170	0.487
Did the exams make you nervous?	0.324	0.134	**0.749**	0.111	0.574
Did the exam hurt?	0.170	-0.010	**0.491**	0.264	0.277
Did you feel tense?	0.381	0.133	**0.795**	0.094	0.656
Were you nervous?	0.335	0.197	**0.767**	0.056	0.627
Were you uncomfortable being partly undressed?	0.183	0.135	0.367	**0.708**	0.561
Were you embarrassed having your private parts?	0.201	0.087	0.280	**0.832**	0.712
How worried are you that you would lose your chance to have a baby?	0.200	**0.339**	0.005	0.271	0.178
Have you been worried that you could give the problem to a sexual partner?	0.263	**0.486**	0.014	0.460	0.408
Have you been worried whether a sexual partner will think they can catch the problems from you?	0.276	**0.833**	0.003	0.127	0.702
Have you been worried whether you should continue having sex?	0.311	**0.678**	0.029	0.219	0.493
Have you been worried that this problem might affect how attractive you are to your sexual partner?	0.207	**0.702**	0.067	-0.051	0.524
Have you been worried whether having sex will make the problem worse?	0.069	**0.652**	0.077	0.221	0.458
Have you been worried whether others think you have had more sexual partners than you should?	0.166	**0.723**	0.146	-0.003	0.555
Have you been worried about sex being more painful now?	0.213	**0.749**	0.097	-0.030	0.590
Have you been worried that this problem might affect how much you enjoy sex?	0.175	**0.675**	0.046	0.372	0.530
How worried are you that cancer will appear in your body?	**0.792**	0.240	0.286	0.112	0.630
Have you worried about the test results?	**0.680**	0.113	0.275	0.227	0.482
Have you worried that you may have cancer?	**0.778**	0.218	0.249	0.136	0.606
How worried are you that you might die?	**0.859**	0.218	0.321	0.138	0.740
Have you been worried that your problem may turn into cancer?	**0.859**	0.217	0.323	0.131	0.739
How worried are you that you might die from cervical cancer?	**0.822**	0.193	0.304	0.079	0.682
% variance	**26.5**	**13.9**	**8.7**	**5.9**	

CDDQ = The Cervical Dysplasia Distress Questionnaire.

## Discussion

Results showed good validity and reliability of the Serbian version of the CDDQ. The findings suggest that the CDDQ can be used for measuring psychological distress among women with abnormal Pap smear results in Serbia.

The appropriate properties of the Serbian CDDQ scale (internal consistency and test-retest reliability, construct validity) were confirmed in the study sample of Serbian women who participated in the national cervical cancer screening program. The Serbian CDDQ showed high internal consistency with high Cronbach α coefficients for all the subscales (α = 0.844–0.913). Shinn and co-authors [10] had reported similar internal consistency scores for all subscales of the original CDDQ scale, for which the Tension and discomfort subscale score was α = 0.86, the Embarrassment subscale score was α = 0.76, the Sexual consequences subscale score was α = 0.85, and the Health consequences subscale score was α = 0.90. Similarly, for low-income and minority women who received an abnormal Pap test result, Breitkopf and co-authors [11] reported two domains of psychological distress, namely Sexual and reproductive consequences (α = 0.85) and Health consequences (α = 0.92). In a sample of Appalachian women [23], internal consistency of the Tension and discomfort subscale was α = 0.83, whereas the Embarrassment subscale score was α = 0.65. Also, in a study that evaluated knowledge of cervical dysplasia and human papilloma virus, the scores of two CDDQ subscales, namely Embarrassment and Discomfort regarding the procedures, demonstrated good internal consistency reliability (α = 0.79 and 0.85 respectively) [24]. Unfortunately, in the available literature there are no data about the psychometric properties of the German version of the CDDQ, the use of which has been reported for women diagnosed with human papilloma virus-related precancerous genital lesions [14]. The item reducing the consistency of the original CDDQ scale [10] was the same in our study: “*How worried are you that you would lose your chance to have a baby*?” Also, one item only (“*How worried are you that you will lose your chance to have a baby*?”) had low communality (0.178) in our study, similar to the value (0.19) from the original study by Shinn and co-authors [10]. Shinn and co-authors [10] suggested that a possible explanation of this low correlation with other items can be that it is the only CDDQ item that deals with problems of reproduction. Results of our study show that reliability of the CDDQ scores in all subscales and in total are satisfactory and similar to those of other surveys in the United States [10,23,24]. Except for our study, no other reported study has been conducted to assess test–retest reliability of the CDDQ scale. In our study, the test-retest reliability ranged from 0.805 to 0.893, indicating that there is a good test-retest reliability of the CDDQ scale. The present study demonstrates that CDDQ scale measures psychological distress consistently over time. Although test-retest showed good reliability, small variability in two time points could be due to the differences in two observations which were not performed under exactly identical conditions, or recall bias, or due to true changes which have occurred between the first and repeated estimation.

Factor analyses in our sample of Serbian women showed that the translated and culturally-adapted CDDQ scale consisted of four main factors, which explained 55.0% of total variance (Health consequences: 26.5%, Sexual and reproductive consequences: 13.9%, Tension and discomfort: 8.7%, and Embarrassment: 5.9%). Shinn et al. [10] divided items into two sets and conducted factors analysis separately, with one set referring to distress related to the medical procedures (including the Tension and discomfort and Embarrassment subscales, respectively accounting for 42.9% of the variance and for an additional 11.5% of the variance) and the other addressing perceived consequences (including the Reproductive and sexual consequences and Health consequences subscales, respectively accounting for 36.7% of the variance and for an additional 11.4% of the variance) of receiving an abnormal Pap smear result. Additionally, we conducted the same factor analysis (with Alpha Factoring) separately, such as was done in the original study: firstly, for distress during medical procedures (including the Tension and discomfort and Embarrassment subscales) and, after that, for the distress related to the consequences (including Reproductive and sexual consequences and Health consequences). We obtained results very similar to those of the original study (the results are not shown to stay within our focused aim to show validity of the Serbian CDDQ scale as a whole). It is important to note that one study alone does not prove construct validity of an instrument, and it is necessary to evaluate evidence from numerous studies using the instrument. However, apart from the findings of the original study by Shinn and co-authors [10] and our results, no other studies have published data on the validity of the used instrument, i.e. the CDDQ scale.

Results from all of the Serbian CDDQ subscales showed fewer disagreements in the assessment of the dimension in comparison to the reference population of American women who had received an abnormal Pap test result [10]. Possible explanations for the observed differences in the findings between the studies include differences in the demographic characteristics of the participants by age, level of education, marital status, etc. On the other hand, some of our results have confirmed some findings from other studies that have applied a different methodology [6].

Only the original study evaluated the factor structure of the CDDQ scale [10] and found a four factor structure, as confirmed in our study. Due to the their specific aims, other studies applied methodology which involved omission of certain subscales of the CDDQ scale and, therefore, these studies reported data on only two [23, 24] or three subscales [14]. Certainly, future studies using the CDDQ scale are necessary to better understand its psychometric properties among women with abnormal Pap smear results.

### Strengths and limitations of the study

According to our best knowledge, this is the first validation study of the Serbian version of the CDDQ scale. Also, based on the available literature, it is the second validation study of the CDDQ scale in the world. The main strengths of this study lie in its assessments of both reliability (internal consistency and test-retest) and validity. Further, the participation rate in our study was satisfactory, at 81.1%. But, our study also has several limitations. In addition to the known drawbacks of cross-sectional studies, a limitation of this study is its sample size. Of course, the question of representativeness of the sample always exists, either because of the barriers in engaging the participants for research due to fear of breach of confidentiality or because the sample was from a country with limited resources, where this sample may be the consequence of both low coverage and compliance of the participants in the screening program for cervical cancer itself. Finally, all results were based on self-reported data, which could be subject to information bias.

## Conclusion

The Serbian version of the CDDQ scale is a valid and reliable instrument for the assessment of psychological distress among women with abnormal Pap smear results. Identification of good validity and reliability of this scale among Serbian women expands the overall understanding and improves the evidence base for use of the CDDQ scale in cross-cultural research. Finally, good validity and reliability of the CDDQ allow for the application of this questionnaire to achieve accurate assessment of the psychological impact of cervical screening and a deeper understanding of individuals across different types of cultures.

## Supporting information

S1 File STROBE checklist. (DOC)

## References

[1] Vaccarella S, Franceschi S, Engholm G, Lönnberg S, Khan S, Bray F. 50 years of screening in the Nordic countries: quantifying the effects on cervical cancer incidence. Br J Cancer. 2014;111:965–9. 10.1038/bjc.2014.362 24992581 PMC4150271

[2] Ministry of Health, Republic of Serbia. National Health survey, Serbia 2013. Belgrade, Serbia: Ministry of Health, Republic of Serbia, 2014.

[3] Mlange R, Matovelo D, Rambau P, Kidenya B. Patient and disease characteristics associated with late tumour stage at presentation of cervical cancer in northwestern Tanzania. BMC Womens Health. 2016;16:5 10.1186/s12905-016-0285-7 26809986 PMC4727267

[4] Mwaka AD, Garimoi CO, Were EM, Roland M, Wabinga H, Lyratzopoulos G. Social, demographic and healthcare factors associated with stage at diagnosis of cervical cancer: cross-sectional study in a tertiary hospital in Northern Uganda. BMJ Open. 2016;6:e007690 10.1136/bmjopen-2015-007690 26801459 PMC4735146

[5] Hui SK, Miller SM, Wen KY, Fang Z, Li T, Buzaglo J et al Psychosocial barriers to follow-up adherence after an abnormal cervical cytology test result among low-income, inner-city women. J Prim Care Community Health. 2014;5:234–41. 10.1177/2150131914529307 24718518 PMC4169747

[6] Gray NM, Sharp L, Cotton SC, Masson LF, Little J, Walker LG et al; TOMBOLA group. Psychological effects of a low-grade abnormal cervical smear test result: anxiety and associated factors. Br J Cancer. 2006;94:1253–62. 10.1038/sj.bjc.6603086 16622462 PMC2361408

[7] Mirowsky J, Ross CE. Measurement for a human science. J Health Soc Behav. 2002;43:152–70. 12096697

[8] Fylan F. Screening for cervical cancer: a review of women's attitudes, knowledge, and behaviour. Br J Gen Pract. 1998;48(433):1509–14. 10024713 PMC1313202

[9] Bennetts A, Irwig L, Oldenburg B, Simpson JM, Mock P, Boyes A, et al PEAPS-Q: a questionnaire to measure the psychosocial effects of having an abnormal pap smear. Psychosocial Effects of Abnormal Pap Smears Questionnaire. J Clin Epidemiol. 1995;48(10):1235–43. 10.1016/0895-4356(95)00015-v 7561985

[10] Shinn E, Basen-Engquist K, Le T, Hansis-Diarte A, Bostic D, Martinez-Cross J et al Distress after an abnormal Pap smear result: scale development and psychometric validation. Prev Med. 2004;39:404–12. 10.1016/j.ypmed.2004.02.004 15226053

[11] Breitkopf CR, Dawson L, Grady JJ, Breitkopf DM, Nelson-Becker C, Snyder RR. Intervention to improve follow-up for abnormal Papanicolaou tests: a randomized clinical trial. Health Psychol. 2014;33:307–16. 10.1037/a0032722 23730719 PMC4025914

[12] Milenkovic M. Analysis of the results of opportunistic and organized cervical cancer screening in the Belgrade municipality of Čukarica Doctoral Dissertation. Belgrade, Serbia: Faculty of Medicine, University of Belgrade, 2016.

[13] World Health Organization. Global Health Observatory data repository. Cervical cancer screening Response by country. Geneva, Switzerland: World Health Organization, 2018.

[14] Nagele E, Reich O, Greimel E, Dorfer M, Haas J, Trutnovsky G. Sexual Activity, Psychosexual Distress, and Fear of Progression in Women With Human Papillomavirus-Related Premalignant Genital Lesions. J Sex Med. 2016;13:253–9. 10.1016/j.jsxm.2015.12.012 26782607

[15] Ilic M, Ilic I. Cancer mortality in Serbia, 1991–2015: an age-period-cohort and joinpoint regression analysis. Cancer Commun (Lond). 2018;38(1):10 10.1186/s40880-018-0282-3. 29764495 PMC5993142

[16] Ministry of Health of Serbia. The national program for early detection of cervical cancer. "Official Gazette of RS", No. 73, 2013 and 83, 2013 Belgrade, Serbia: Ministry of Health of Serbia, 2013.

[17] Kesić V, Sparić R, Watrowski R, Dotlić J, Stefanović R, Marić G et al Cross-cultural adaptation and validation of the Functional Assessment of Chronic Illness Therapy—Cervical Dysplasia (FACIT-CD) questionnaire for Serbian women. Eur J Obstet Gynecol Reprod Biol. 2018;226:7–14. 10.1016/j.ejogrb.2018.05.009 29777860

[18] Mundfrom DJ, Shaw DG & Ke TL. Minimum sample size recommendations for conducting factor analysis. International Journal of Testing. 2005;5:159–68.

[19] MacCallum RC, Widaman KF, Zhang S & Hong S. Sample size in factor analysis. Psychol Methods. 1999;4(1):84–99.

[20] Institute of Public Health of Serbia. Cervical cancer screening. Belgrade, Serbia: Institute of Public Health of Serbia, 2018.

[21] Beaton DE, Bombardier C, Guillemin F, Ferraz MB. Guidelines for the process of cross-cultural adaptation of self-report measures. Spine (Phila Pa 1976). 2000;25:3186–91. 11124735 10.1097/00007632-200012150-00014

[22] Ebel RL & Frisbie DA. Essentials of educational measurement. Fifth edition Englewood Cliffs, NJ: Prentice-Hall, 1991.

[23] Fortner KB, Zite NB, Wallace LS. In my own words: misunderstanding of Pap smears and colposcopy among Appalachian women. J Low Genit Tract Dis. 2007;11:251–7. 10.1097/LGT.0b013e318033999f 17917569

[24] Pruitt SL, Parker PA, Peterson SK, Le T, Follen M, Basen-Engquist K. Knowledge of cervical dysplasia and human papillomavirus among women seen in a colposcopy clinic. Gynecol Oncol. 2005;99(3 Suppl 1):S236–44. 16150483 10.1016/j.ygyno.2005.07.095

